# Digital health literacy among primary and secondary school teachers—a quantitative study

**DOI:** 10.3389/fpubh.2024.1334263

**Published:** 2024-06-07

**Authors:** Pia Rangnow, Lisa Fischer, Anja Hartmann, Denise Renninger, Lisa Stauch, Orkan Okan, Kevin Dadaczynski

**Affiliations:** ^1^Department of Health Sciences, Fulda University of Applied Sciences, Fulda, Germany; ^2^Department of Applied Health Sciences, Hochschule für Gesundheit, University of Applied Sciences, Bochum, Germany; ^3^Department of Physiotherapy, Institute of Health Sciences, University of Lübeck, Lübeck, Germany; ^4^Department of Health and Sport Science, TUM School of Medicine and Health, Technical University of Munich, Munich, Germany; ^5^WHO Collaborating Centre for Health Literacy, TUM School of Medicine and Health, Technical University of Munich, Munich, Germany; ^6^Center for Applied Health Sciences, Leuphana University Lueneburg, Lueneburg, Germany

**Keywords:** digital health literacy, teachers, health promotion, health information, prevention, health education, determinants

## Abstract

**Introduction:**

Digital health literacy (DHL) is a key competency for individuals’ daily decisions toward their health behavior and wellbeing. While there is much focus on health literacy (HL) among the general population, teachers have been rarely addressed. Given the shortages in the teaching workforce in Europe and the impact of demanding working conditions on their health, it is important to address DHL in teachers. This paper examines the DHL of primary and secondary teachers and its associations with sociodemographic and school-related factors.

**Methods:**

An online cross-sectional study was conducted with 1,600 German primary and secondary school teachers between October and December 2022. To assess DHL, the Digital Health Literacy Instrument (DHLI) including seven subscales was used. Statistical analyses were conducted on item and subscale level and an overall DHL score was calculated. Next to descriptive analyses, bivariate and regression analyses were conducted to explore potential associations with sociodemographic and school-related factors.

**Results:**

The frequency of difficulty in using digital health information varied across DHL dimensions and was greatest for *protecting privacy* (70.9%) and *evaluating reliability* (40.0%). In multivariate analysis, females more often reported a sufficient ability of *adding content* (OR = 1.61, CI = 1.05–2.48), while males more often reported a sufficient ability to *protect their privacy* (OR = 0.45, CI = 0.27–0.75). Teachers with leadership positions more often reported a sufficient ability in *adding content* (OR = 1.78, CI = 1.07–2.98). Regarding the ability to *determine the relevance* of online health-related information, no associations with a predictor variable were found.

**Discussion:**

The results suggest that it is important to examine the individual dimensions of DHL and their distinct associations with sociodemographic and school-level factors, rather than just to rely on the overall level of DHL. The differential patterns identified in this study suggest a greater intervention need for teachers from higher age groups, primary and secondary general schools, and those without leadership roles. However, based on the limited predictive power of the variables included, further individual and school-level factors and their potential association with DHL should be investigated in the future. The promotion of DHL should be integrated into both teacher education and in-service training.

## 1 Introduction

Modern societies and everyday realities are increasingly characterized by a growing diversity of media and information, including the various ways to access and navigate them. The consequences of this development became clear during the COVID-19 pandemic, when people had to use digital and online services more and more frequently. Among other things, the infodemic, an overabundance of reliable and incorrect, trustworthy, and untrustworthy information on the Internet, was just one of the many characteristics (1). Strengthening digital and general health literacy (HL) is seen as one of the pillars in the fight against the infodemic (2). Furthermore, digital health literacy (DHL) is a key competence for individuals to make informed everyday decisions to improve or maintain their health, well-being and thus quality of life (3–6). DHL refers to the ability to find health-related information from electronic resources and to deal with it appropriately, to promote and maintain health or handle health problems (7). In addition to the general HL core abilities of finding, understanding, assessing, and using information, DHL also includes the ability to communicate and handle information securely (i.e., data protection) (8, 9). Therefore, it is fitting for a more collaborative information environment, often called Health 2.0 (5), where interaction skills are required to communicate with healthcare providers, peers or wider audiences about health, engage with health-related content on social media or receive treatment and support over the internet. The improvement of general digital literacy is promoted in the EU through the Digital Competence Framework for Citizens (DigComp) to develop the population’s ability to deal with misinformation and disinformation, to interact with AI systems or emerging technologies and to work well in new contexts such as remote or hybrid work (10).

From a theoretical perspective, Sørensen et al. (11) and van der Vaart and Drossaert (5) proposed integrated conceptual models on general and digital HL suggesting that an increased ability in information management and applying technological skills contributes to healthier behaviors and better health outcomes. In addition, the conceptual models include personal and situational determinants to the above equation, such as income, education, age, sex, employment and physical environment and their resulting demands (5, 11). A relational concept of health literacy is assumed, in which individual-level factors and demands of the systems interact and determine health literacy (12). A recent representative study indicates that only 24.2% of the German adult population have a sufficient DHL (13) and that a limited DHL is associated with increasing age, low education levels, low socioeconomic status, as well as low functional literacy or financial resources (9, 13).

As research on DHL is relatively new, there are only limited study findings on the extent and socio-demographic differences, which often focus on specific population groups, e.g., university students (14–18). Therefore, in order to identify relevant socio-demographic factors, the much broader body of research on general HL must also be taken into account.

As a social determinant of health, the level of general and digital HL depends on the individual’s context (19), their access to education or to community opportunities, so that a person’s general and digital HL is determined by the factors of the systems with which they interact (20). A given digital infrastructure can either support or impede a person in coping with the complexity and demands of the digital space and schools are places where general and digital HL levels can be promoted (20). Moreover, DHL is considered a ‘super’ social determinant of health (21) as it has implications for other social determinants, such as how an individual uses the health care system offerings or accesses social assistance programs that are increasingly, sometimes exclusively, available online (21, 22), thus, influencing the level to which an individual can meaningfully participate in modern society (22). It can be assumed that a sufficient general and digital HL serves not only the individual but also those in the immediate social environment and society in general (12, 23, 24). Thus, as an individual with a sufficient level of HL it is possible to take responsibility for improving the health (literacy) of others (12), e.g., by changing learning conditions in the school setting (26).

So far, research on HL in the school setting focused mainly on pupils (8, 26, 27), while teachers have hardly been in focus (4, 28, 29). This is surprising, as many European countries - including Germany - experience a shortage of teachers (30, 31), which could be attributed to the fact that numerous studies have pointed to stressful working conditions (4, 32–34). These are risk factors for health such as burnout, somatic symptoms such as headaches, or cardiovascular diseases (32). Sufficient levels of DHL are thus an important determinant of teacher’s health (35). A teacher’s health can be regarded as an important resource for the overall school climate and quality of teaching (36–40) and their well-being correlates among other things with better student academic performance (8, 41, 42). Moreover, study findings indicate that teacher’s HL serves as a significant predictor of the level of implementation of health promotion activities in school (41, 43–46), the same can be assumed for DHL. To date, there have been no studies that have examined the DHL of teachers, there are, however, studies on general HL. In a study with 680 German school principals, almost 30% showed a limited HL, with most difficulties found in assessing the reliability of health information (4). In other studies, from Sri Lanka, Iran and Germany, the proportion of teachers with problematic and inadequate HL ranged from 32.5 to 50.6% (29, 46, 47).

The literature reports differences in general and digital HL by individual-level factors such as sex, age or migration background for teachers and the general population although the available study findings mainly relate to general HL (4, 29, 47–50) and are not homogeneous. Regarding the differences of teachers’ sex on the level of HL, previous research has shown that female teachers with and without leadership positions tend to have higher levels of HL than their male counterparts (4, 29, 48). Regarding age, two studies indicated that a lower age of teachers was associated with higher HL. In a sample of teachers from Sri Lanka (47) it was found that a lower age (45 years or less) was associated with higher levels of limited HL. By contrast, in another study with Turkish teachers, a higher proportion of respondents with adequate HL was observed for younger respondents (25 to 34 years) (48). This finding is also reflected in a systematic review and meta-analysis of DHL that found a negative association of increasing age on DHL levels among general adult populations (49). In contrast, a study among German school principals found no age differences (4). Regarding migration background, a German-wide population-based study found that people with an own migration background hardly differ from the population average without a migration background in terms of DHL, while respondents with a parental migration background had a higher DHL level than people without a migration background (11). In terms of school-related characteristics, existing studies did not report any difference for type of school (4, 50) or teacher’s position at school (4, 50). As previous empirical evidence is mainly limited to their general HL it is important to examine the level of DHL among teachers and determine whether and how it differs regarding sociodemographic or school-level factors. Drawing on the existing conceptual models mentioned above and the available findings from previous research, this paper addresses the following two research questions:

1. What is the state of digital health literacy among primary and secondary teachers?
2. What sociodemographic and school-related factors are associated with primary and secondary teachers’ digital health literacy?

## 2 Materials and methods

### 2.1 Study design

To examine the DHL of primary and secondary teachers in Germany, an online cross-sectional survey in all 16 German federal states was conducted from October to December 2022. Following an ethical approval by the ethics committee of Fulda University of Applied Sciences (reference number 3.1.9.2), eligible study participants were recruited via a communication campaign run by a German agency for educational communication through print and digital communication channels. Participation was voluntary and anonymity was ensured, informed consent was given before participation. Postal invitations and reminders 3 weeks after the initial invitation were sent to 5,000 teachers. Those teachers were randomly selected, stratified according to school type and federal state and contacted by the agency. Furthermore, an advertisement in a printed teachers’ magazine with a circulation of 80,000 copies served to disseminate the survey. It was also disseminated through online media such as eight teacher-specific newsletters, five social media channels, five online commercials and content ads on educational websites for teachers, each with a reach of between 6,000 and 28,600 recipients approximately. To increase the motivation to participate, incentives were awarded as 25 vouchers worth 100 euros each, which could be redeemed at any time at more than 500 shops. To prevent the collection of individual contact data, the raffle was conducted via the external German agency for educational communication.

### 2.2 Measures

Sex, age, and migration background were used as sociodemographic variables for the purpose of this study. Sex was operationalized using female, inter*/intergender, male as categories, while age was assessed as open information and subsequently categorized (<40, 40–49, 50–59, 60+). Migration background was calculated using the procedure of the Health Behavior in School-aged Children (HBSC) study: (50) one-sided (i.e., one parent of respondent(s) was not born in Germany), (1) two-sided (i.e., respondent/s themselves were not born in Germany and one parent was not born in Germany or both parents were not born in Germany), and (2) no migration background (51). School-level factors included the position of the teachers, i.e., teachers with or without a leadership function, and the type of school they worked at. Teachers with a leadership position means that they held the role of principal or were part of the school management team.

The Digital Health Literacy Instrument (DHLI) by van der Vaart and Drossaert (5) was used in this study, reflecting the complexity of Health 2.0 environments through differentiating seven DHL dimensions. Each dimension captures different facets of an individual’s ability to access (*operational skills, navigation skills, information searching*), process (*evaluating reliability, determining relevance, protecting privacy*), and communicate with or about digital health information (*adding content*). Following van der Vaart and Drossaert, the instruction was given, that if respondents do not post messages on social media or other channels, they could skip all items of the subscales *adding content* and *protecting privacy* (5). Each subscale includes three items that could be answered on a four-point scale (1 = very difficult to 4 = very easy or 4 = never to 1 = often). As previous studies revealed a low reliability for the subscale *protecting privacy* (4, 5), two items were added to this dimension, namely “Do you find it difficult to determine how the security of your private data is ensured by the media provider?” and “Do you find it difficult to determine who has access to your data?.” In this study the internal consistency (Cronbach α) of the six subscales ranged from acceptable to excellent (0.72 < α < 0.90). The extended *protecting priv*acy subscale reached a good reliability (α = 0.82).

### 2.3 Statistical analyses

#### 2.3.1 Calculating subscale scores

In a first step, a sum score was calculated for each subscale under the condition that all items were answered. This sum scores ranged from 5 to 20 for *protecting privacy* (5 items) and 3 to 12 for the other four subscales with three items, with higher values reflecting a higher DHL level. In a second step, the sum score was grouped into three categories according to previous research (8). Participants who predominantly rated all items as “very difficult” or “difficult” (inadequate ability in this dimension = 3 to 6 resp. 5 to 10), participants who predominantly rated the items as “very easy” or easy (sufficient ability in this dimension = 9 to 12 resp. 15 to 20) and those which fell between these two categories (problematic ability in this dimension = 7 to 8 resp. 11 to 14).

#### 2.3.2 Calculating an overall score

Five of the seven subscales (*operational skills, navigation skills, information searching, evaluating reliability, and determining relevance*) were used to calculate an overall DHL sum score, that ranged from 5 to 15. Two scales (*adding content* and *protecting privacy*) were not included here because the proportion of missing values was high (60.5 and 29.0% of respondents, respectively) due to the instructions given. Based on previous work (8), in a second step the following cut-off values were then applied to categorize the range of 5 to 15: 5 to 7 = 1 (inadequate DHL), 8 to 12 = 2 (problematic DHL), and 9 to 15 = 3 (sufficient DHL).

#### 2.3.3 Bivariate and multivariate analysis

Following a descriptive data analysis, we conducted bivariate analyses to test for significant differences between the three categories of DHL and sociodemographic and school-level factors, using cross tabulation with subsequent chi-square test (χ^2^). In cases with cell frequencies below 5 an exact Fisher test (FET) was performed. If no 2 × 2 cross-tabulation was given, the Monte Carlo simulation was performed. The strength of the association was determined using the Cramer index (Cramer V) and Phi Index (φ). According to Cohen (52), the strength of each association was interpreted as an effect size measure using the following conventions: ≥0.10 (small), ≥0.30 (medium), ≥0.50 (large).

In a final step, six binary logistic regressions analyses were performed to determine the associations of all explanatory variables (sex, age, migration background, position, and type of school) with the DHL subscales (*navigation skills, information searching, evaluating reliability, adding content and protecting privacy*) by odds ratio (OR) and its respective 95% confidence interval (95% CI). For this step, problematic and inadequate DHL were summarized in the category limited DHL. *Operational skills* was omitted from further analysis because responses were very homogeneous and indicated that the majority of respondents (99.3%) experienced no difficulties, which is why the variance across categories and predictors was low (see 3.2). According to the correlation matrix, multicollinearity was not a confounding factor in the analysis (r > 0.80) (53). Cases with student residuals of more than ±3 standard deviations were considered outliers (54), but none were found in all six regression models. To measure the goodness of fit of the six regression models we used the Hosmer-Lemeshow Test. For variance explanation we used Nagelkerkes R^2^, effects can be interpreted as follows: *R*^2^ > 0.20 small effect, *R*^2^ > 0.40 medium effect, *R*^2^ > 0.50 high effect (55). To determine the goodness of classification, we used the percentage of accuracy in classification (PAC) in the confusion matrix (see Table 1). Explanatory variables were included in the regression models based on existing empirical findings highlighted in the introduction (for sex, age, migration background, type of school) or based on the bivariate results (position). For all analyses, the significance level was set at *p* = 0.05. All calculations were performed using IBM SPSS Statistics 28 statistical software (IBM Corp., Armonk, NY, United States).

**Table 1:** Characteristics of regression models.

	NAV**	IS	AC	ER	DR	PP
Model significance χ^2^	χ^2^ (11) = 30.942, *p* < 0.01	χ^2^ (11) = 13.353, n.s.	χ^2^ (11) = 11.842, n.s.	x^2^ (11) = 10.032, n.s.	χ^2^ (11) = 10.941, n.s.	χ^2^ (11) = 19.304, n.s.
N of model	1,258	1,258	775	1,255	1,251	369
Nagelkerkes *R*^2^	*R*^2^ = 0.040	*R*^2^ = 0.015	*R*^2^ =0.025	*R*^2^ = 0.011	*R*^2^ =0.012	*R*^2^ =0.071
Hosmer-Lemeshow test	χ^2^ (8) = 11.048, *p* = 0.199	χ^2^ (8) = 3.537, *p* = 0.896	χ^2^ (8) = 7.378, *p* = 0.496	χ^2^ (8) = 2.265, *p* = 0.972	χ^2^ (8) = 6.201, *p* = 0.625	χ^2^ (8) = 10.600, *p* = 0.225
Accuracy in classification (Sensitivity % and Specificity %)	82.2% (0 and 100%)	68% (0 and 100%)	82.6% (0 and 100%)	54.9% (91.7 and 10.5%)	56.5% (91% and 11,1%)	65.6% (91.1 and 13.9%)

**p < 0.01; n.s., not significant; NAV, navigation skills; IS, information searching; AC, adding content; ER, evaluating reliability; DR, determining relevance; PP, protecting privacy.

## 3 Results

### 3.1 Sample characteristics

After a data check for consistency and plausibility, the final sample consisted of 1,600 respondents. As shown in Table 2, 74.6% of participants reported being female, 25.4% were male, while one person who stated to be inter*/intergender was excluded from bivariate and multivariate analyses due to the small number of cases. In terms of age, participants in the 50–59 age group were most represented in the sample (36.0%), while older teachers (60+) with 11% made up the smallest proportion. Due to the small number of cases migration background was dichotomized into teachers with and without migration background. 88.3% of teachers indicated that they had no migration background. Almost half of the respondents worked in (vocational) grammar schools (47.8%), while only 3.3% reported intermediate schools as their place of work. In comparison with the total number of teachers in Germany, there are certain sociodemographic similarities to our sample. For example, in 2022 there were 73.6% female and 26.0% male teachers (this study 74.5% female, 25.4% male) in Germany (56). While those under 40 were underrepresented in this study by 11.0%, those aged 50–59 were overrepresented in this study by 10.2%. Teachers aged 40–49 and 60+ were similarly represented in the study and in the German teaching population at around 27.0% (57).

**Table 2:** Characteristics of study sample (*n* = 1,295–1,600).

	*n*	*%*
**Sex**		
Inter* / Intergender	1	0.1%
Female	1,193	74.5%
Male	406	25.4%
**Age**		
< 40	405	25.4%
40–49	444	27.8%
50–59	572	35.8%
60+	176	11.0%
**Migration background**		
One-sided	99	6.2%
Two-sided	88	5.5%
No	1,407	88.3%
**Leadership position**		
Yes	380	24.3%
No	1,183	75.7%
**School type**		
Primary school	114	8.8%
Secondary general school	39	3.0%
Intermediate school	187	14.4%
(vocational) grammar school	565	43.6%
Integrated comprehensive school	197	15.2%
School with several levels of qualification	193	14.9%
**Total**	**1,600**	**100%**

### 3.2 Level of digital health literacy

While for 60.7% of teachers a sufficient level of DHL was observed, 37.9% showed a problematic and 1.3% an inadequate level of DHL. Table 3 shows the percentage of respondents who reported difficulties in each of the 23 DHL items (“difficult/very difficult” or “sometimes/often”). Reported difficulties ranged from 0.4% for one *operational skills* item (“do you find it difficult to use the mouse”) to 70.9% for one *protecting privacy* item (“do you find it difficult to determine who has access to your data”). This subscale also contained the second most difficult item, with 67.5% stating that they find it (very) difficult to assess how the security of their private data is ensured by the media provider. Thus, the highest average difficulty was found for the dimension *protecting privacy* with 47.0%, followed by *evaluating reliability* with 40.0% and *determining relevance* with 39.0%. *Operational* and *navigation skills* posed the least difficulties for respondents (0.53% and 16.2% respectively).

**Table 3:** Frequency of the response option “difficult/very difficult” of the individual items on digital health literacy (*n* = 487–1,600).

No.	Item	% (n)	95%-CI
**Operational skills**			
1	… use the keyboard of a computer, tablets or smartphones (e.g., to type words)? (N=1,590)	0.6 (10)	0.3–1.0
2	… use the mouse (e.g., to put the cursor in the right field or to click)? (N=1,589)	0.4 (7)	0.2–0.6
3	… use the buttons or links and hyperlinks on websites? (N=1,586)	0.6 (9)	0.3–0.9
**Navigation skills**			
4	... you lose track of where you are on a website or the Internet? (N=1,369)	14.3 (229)	12.7-19.0
5	… you do not know how to return to a previous page? (N=1597)	5.4 (87)	4.4-6.6
6	… you click on something and get to see something different than you expected? (N=1149)	28.0 (447)	25.9-30.3
**Information searching**			
7	… make a choice from all the information you find? (N=1249)	21.9 (350)	20.0-23.6
8	… use the proper words or search query to find the information you are looking for? (N=1382)	13.6 (218)	12.1-15.4
9	… find the exact information you are looking for? (N=1143)	28.3 (451)	26.0-30.05
**Adding content**			
10	… clearly formulate your question or health-related worry? (N=892)	9.8 (97)	8.1-11.6
11	… express your opinion, thoughts, or feelings in writing? (N=879)	12.4 (124)	10.6-14.2
12	… write your message as such, for people to understand exactly what you mean? (N=883)	12.0 (120)	12.0-13.9
**Evaluating reliability**			
13	… decide whether the information is reliable or not? (N=887)	44.5 (710)	42.0-46.8
14	… express your opinion, thoughts, or feelings in writing? (N=850)	46.8 (747)	44.8-49.1
15	… write your message as such, for people to understand exactly what you mean? (N=1135)	28.8 (458)	26.5-31.1
**Determining relevance**			
16	… use the information you found to make decisions about your health (e. g., on nutrition, medication or to decide whether to ask a doctor’s opinion)? (N=1086)	32.0 (510)	29.9-34.2
17	… apply the information you found in your daily life? (N=928)	41.7 (665)	39.2-44.3
18	… decide if the information you found is applicable to you? (N=903)	43.4 (691)	40.9-45.6
**Protecting privacy**			
19	… do you find it difficult to judge who can read along? (N=234)	52.0 (253)	47.8-56.1
20	… do you find it difficult to determine, how the security of your private data is guaranteed by the media provider? (N=165)	67.5 (342)	63.5-71.2
21	… do you find it difficult to determine, who has access to your data? (N=147)	70.9 (359)	67.2-74.5
22	… do you (intentionally or unintentionally) share your own private information (e. g., name or address)? (N=335)	34.7 (178)	31.0-28.6
23	… do you (intentionally or unintentionally) share some else’s private information? (N=441)	14.4 (74)	11.7-17.4

N, total number of cases; n, number of cases responded with “difficult/very difficult” resp. “sometimes/often” for the subscale protecting privacy, CI confidence interval.

### 3.3 Sociodemographic and school-level differences of digital health literacy

Stratified by sociodemographic and school-level factors, the results of the bivariate analyses revealed significant differences for five of seven DHLI dimensions (see Table 4). Educators aged younger than 40 years most often reported sufficient *navigation skills*, while those aged 50–59 years were found to report the most difficulties (χ^2^(6) = 19.30, *p* < 0.01). Female respondents significantly more often reported a sufficient ability *to search for information* (FET = 8.79, *p* < 0.05) and to *determine the relevance* of health information retrieved (FET = 12.20, *p* < 0.01). Respondents with a two-sided migration background stated more frequently that they were able to *protect their and others privacy* sufficiently, compared to respondents without a migration background and respondents with a one-sided migration background (χ^2^(4) = 13.86, *p* < 0.01). Teachers with leadership position more often showed a sufficient ability to *add content* than respondents without leadership position (χ^2^(2) = 6.63, *p* < 0.05).

**Table 4:** Digital health literacy stratified by sociodemographic and school-related factors.

	Operational skills			Navigation skills			Information searching			Adding content		
	*sufficient % (n)*	*problematic % (n)*	*inadequate % (n)*	*sufficient % (n)*	*problematic % (n)*	*inadequate % (n)*	*sufficient % (n)*	*problematic % (n)*	*inadequate % (n)*	*sufficient % (n)*	*problematic % (n)*	*inadequate % (n)*
**Sex**	n.s.			n.s.			FET = 8.79, *p <* 0.05, φ *= 0*.137			n.s.		
Female	99.6 (1180)	0.3 (4)	0.1 (1)	81.0 (961)	14.6 (173)	4.5 (53)	69.1 (820)	21.7 (257)	9.2 (109)	83.7 (595)	11.4 (81)	4.9 (35)
Male	98.5 (399)	1.0 (4)	0.5 (2)	81.7 (221)	13.1 (53)	5.2 (21)	63.5 (258)	22.7 (92)	13.8 (56)	78.1 (207)	14.3 (38)	7.5 (20)
**Age**	n.s.			χ^2^(6) = 19.30*, p* < 0.01, V = 0.003			n.s.			n.s.		
< 40	99.3 (401)	0.5 (2)	0.2 (1)	86.1 (348)	12.4 (50)	1.5 (6)	68.6 (278)	22.0 (89)	9.4 (38)	77.3 (191)	16.2 (40)	6.5 (16)
40–49	99.3 (439)	0.7 (3)	0.0 (0)	82.6 (366)	12.6 (56)	4.7 (21)	67.7 (300)	21.7 (96)	10.6 (47)	82.4 (220)	12.7 (34)	4.9 (13)
50–59	99.1 (560)	0.5 (3)	0.4 (2)	77.5 (440)	16.5 (94)	6.0 (34)	66.0 (373)	21.8 (123)	12.2 (69)	84.1 (302)	9.5 (34)	6.4 (23)
60+	100 (176)	0.0 (0)	0.0 (0)	78.2 (136)	14.9 (26)	6.9 (12)	71.6 (126)	22.2 (39)	6.3 (11)	86.1 (87)	10.9 (11)	3.0 (3)
**Migration background**	n.s.			n.s.			n.s.			n.s.		
Yes	98.9 (185)	1.1 (2)	0.0 (0)	81.2 (151)	15.6 (29)	3.2(6)	66.5 (123)	21.1 (39)	12.4 (23)	83.3 (100)	10.0 (12)	6.7 (8)
No	99.4 (1389)	0.4 (6)	0.2 (3)	81.2 (1137)	14.0 (196)	4.9 (68)	67.8 (950)	22.1 (310)	10.1 (142)	82.0 (697)	12.5 (106)	5.5 (47)
**Leadership Position**	n.s.			n.s.			n.s.			χ^2^(2) = 6.63*, p* < 0.05, φ *=* 0.036		
No	99.4 (1170)	0.4 (5)	0.2 (2)	80.8 (954)	14.7 (174)	4.4 (52)	66.7 (785)	22.5 (265)	10.8 (127)	80.4 (571)	13.4 (95)	6.2 (44)
Yes	99.2 (375)	0.5 (2)	0.3 (1)	82.4 (310)	12.2 (46)	5.3 (20)	71.1 (270)	20.3 (77)	8.7 (33)	87.7 (214)	8.2 (20)	4.1 (10)
**Type of school**	n.s.			n.s.			n.s.			n.s.		
Primary school	98.2 (111)	0.9 (1)	0.9 (1)	81.6 (93)	12.3 (14)	6.1 (7)	65.2 (73)	25.0 (28)	9.8 (11)	79.2 (61)	11.7 (9)	9.1 (7)
Secondary general school	100 (38)	0.0 (0)	0.0 (0)	66.7 (26)	23.1 (9)	10.3 (4)	66.7 (26)	23.1 (9)	10.3 (4)	82.8 (24)	10.3 (3)	6.9 (2)
Imtermediate school	100 (184)	0.0 (0)	0.0 (0)	69.4 (129)	20.4 (38)	10.2 (19)	69.4 (12)	20.4 (38)	10.2 (19)	85.3 (99)	9.5 (11)	5.2 (6)
(vocational) grammar school	99.1 (558)	0.5 (3)	0.4 (2)	85.0	9.6 (43)	5.4 (24)	67.0 (377)	22.6 (127)	10.5 (59)	82.8 (275)	13.3 (44)	3.9 (13)
Integrated comprehensive school	100 (195)	0.0 (0)	0.0 (0)	66.0 (130)	21.8 (43)	12.2 (24)	66.0 (130)	21.8 (43)	12.2 (24)	79.8 (95)	14.3 (17)	5.9 (7)
School with several levels of qualification	100 (193)	0.0 (0)	0.0 (0)	75.0 (144)	18.8 (36)	6.3 (12)	75.0 (144)	18.8 (36)	6.3 (12)	82.9 (102)	10.6 (13)	5.0 (36)
**Total**	99.3	0.5 (8)	0.2 (3)	81.2 (1293)	14.2 (226)	4.6 (74)	67.7 (1079)	21.9 (349)	10.4 (165)	82.2 (802)	12.2 (119)	5.6 (55)

	Evaluating reliability			Determining relevance			Protecting privacy		
	*sufficient % (n)*	*problematic % (n)*	*inadequate % (n)*	*sufficient % (n)*	*problematic % (n)*	*inadequate % (n)*	*sufficient % (n)*	*problematic % (n)*	*inadequate % (n)*
**Sex**	n.s.			FET = 12.20, *p* < 0.01, *φ* = 0.007			n.s.		
Female	43.8 (518)	34.4 (407)	21.8 (258)	45.4 (536)	34.0 (401)	20.6 (243)	24.3 (80)	46.2 (152)	29.5 (97)
Male	43.0 (174)	33.1 (134)	24.0 (97)	36.3 (147)	39.5 (160)	24.2 (98)	30.1 (41)	47.8 (65)	22.1 (30)
**Age**	n.s.			n.s.			n.s.		
< 40	45.9 (185)	33.3 (134)	20.8 (84)	41.1 (166)	36.6 (148)	22.3 (90)	24.3 (34)	48.6 (68)	27.1 (38)
40–49	43.9 (194)	35.1 (155)	21.0 (93)	40.9 (180)	37.5 (165)	21.6 (95)	31.9 (45)	45.4 (64)	22.7 (32)
50–59	41.9 (237)	33.7 (191)	24.4 (138)	45.4 (256)	33.3 (188)	21.3 (120)	23.6 (37)	47.1 (74)	29.3 (46)
60+	42.5 (749)	35.1 (61)	22.4 (39)	46.6 (81)	32.8 (57)	20.7 (36)	18.5 (5)	40.7 (11)	40.7 (11)
**Migration background**	n.s.			n.s.			χ^2^(4) = 13.86, *p* = 0.01, V = 0.019		
Yes	48.4 (90)	34.9 (65)	16.7 (31)	49.2 (91)	35.7 (66)	15.1 (28)	35.5 (22)	53.2 (33)	11.3 (7)
No	42.8 (598)	34.0 (475)	23.2 (324)	42.2 (589)	35.3 (493)	22.4 (313)	25.7 (120)	44.6 (179)	29.7 (119)
**Leadership Position**	n.s.			n.s.			n.s.		
Yes	43.3 (507)	33.8 (396)	23.0 (269)	42.5 (498)	35.2 (412)	22.4 (262)	20.2 (23)	56.1 (64)	23.7 (27)
No	45.0 (171)	35.0 (133)	20.0 (76)	44.2 (167)	36.8 (139)	19.0 (72)	28.0 (94)	43.5 (146)	28.6 (96)
**Type of school**	n.s.			n.s.			n.s.		
Primary school	35.7 (40)	35.7 (40)	28.6 (32)	43.0 (49)	38.6 (44)	18.4 (21)	29.2 (14)	45.8 (22)	25.0 (12)
Secondary general school	46.2 (18)	33.3 (13)	20.5 (8)	50.0 (19)	28.9 (11)	21.1 (8)	33.3 (4)	41.7 (5)	25.0 (3)
Intermediate school	45.7 (85)	32.2 (60)	22.0 (41)	41.8 (77)	37.5 (69)	20.7 (38)	22.4 (11)	53.1 (26)	24.5 (12)
(vocational) grammar school	46.3 (260)	34.3 (193)	19.4 (109)	42.6 (238)	35.6 (199)	21.8 (122)	27.8 (42)	39.1 (59)	33.1 (50)
Integrated comprehensive school	48.5 (95)	31.6 (62)	19.9 (39)	43.9 (86)	35.7 (70)	20.4 (40)	30.6 (19)	45.2 (28)	24.2 (15)
School with several levels of qualification	45.3 (87)	34.9 (67)	19.8 (38)	45.0 (86)	35.6 (68)	19.4 (37)	24.6 (15)	55.7 (34)	19.7 (12)
**Total**	43.6 (693)	34.0 (541)	22.3 (355)	43.1 (683)	35.4 (462)	21.5 (341)	26.0 (121)	46.7 (217)	27.3 (127)

n, frequency; n.s., not significant; χ^2^, chi-square; FET, exact test according to Fisher; V, Cramer Index (Cramer V) and φ, Phi Index; subscales categorized into sufficient, problematic and inadequate ability in the respective dimension: sufficient ability in this dimension 9–12, problematic ability in this dimension 7–8, inadequate ability in this dimension 3–6.

### 3.4 Associations between sociodemographic, school-level factors and digital health literacy

Finally, to predict the likelihood of a limited DHL by sociodemographic and school-level characteristics, binary logistic regressions were calculated. Despite a lack of significance in the bivariate analyses and although there were no significant associations in previous studies, school type was added as a predictor in the regression analyses, as this is the first study on teachers’ DHL and interaction effects cannot be ruled out. For *navigation skills* the binary logistic regression model was significant, although the level of sensitivity and specificity indicate a limited explanatory power (see Table 1). All other models were not significant and can only explain a small amount of the variation of the dependent variable by the explanatory variable, but all had appropriate model quality as indicated by the Hosmer-Lemeshow-Test (see Table 1).

The results of the binary logistic regressions indicate that *determining relevance* is not associated with any of the predictor variables (see Table 5). Two significant associations between sociodemographic factors and DHL were found: Being in the 40–49, 50–59 and 60+ age group was associated with an increased likelihood of limited *navigation skills* (OR = 1.60, *p* < 0.01), (OR = 2.17, *p* < 0.01) and (OR = 1.79, *p* < 0.05), respectively. Male sex was found to be associated with an increased likelihood of a limited ability of *adding content* (OR = 1.16, *p* < 0.05) and a lower likelihood of having a limited ability to *protect one’s own and other’s data* (OR = 0.45, *p* < 0.01). Moreover, associations with both school-level factors were found: First, being a teacher without a leadership position was associated with an increased likelihood of a limited ability of adding content (OR = 1.78, *p* < 0.05). Second, a decreased likelihood of a limited ability to *evaluate reliability* of health-related information was observed for teachers from intermediate schools (OR = 0.58, *p* < 0.05), (vocational) grammar schools (OR = 0.58, *p* < 0.05), integrated comprehensive schools (OR = 0.58, *p* < 0.05), and schools with multiple educational programs (OR = 0.60, *p* < 0.05). Furthermore, teachers from schools with multiple educational programs had a significantly lower likelihood of a limited ability of *information searching* (OR = 0.57, *p* < 0.05).

**Table 5:** Binary logistic regression analysis for limited DHL skills.

	Limited NAV *OR (95%-CI)*	Limited IS *OR (95%-CI)*	Limited AC *OR (95%CI)*	Limited ER *OR (95%-CI)*	Limited DR *OR (95%-CI)*	Limited PP *OR (95%-CI)*
**Sex**						
Female (Ref.)	1.00	1.00	1.00	1.00	1.00	1.00
Male	0.87 (0.61–1.25)	1.27 (0.96–1.68)	1.61 (1.05–2.47)*	0.96 (0.73–1.25)	1.21 (0.93–1.59)	0.46(0.28–0.77)**
**Age**						
< 40 (Ref.)	1.00	1.00	1.00	1.00	1.00	1.00
40–49	1.60 (1.04–2.50)*	0.99 (0.71–1.37)	0.78 (0.47–1.30)	1.03 (0.76–1.40)	0.92 (0.69–1.25)	0.65 (0.37–1.15)
50–59	2.32 (1.60–3.44)**	1.21 (0.90–1.65)	0.80 (0.50–1.29)	1.19 (0.88–1.58)	0.88 (0.67–1.19)	1.14 (0.65–2.00)
60+	1.90 (1.10–3.30)*	0.98 (0.63–1.53)	0.96 (0.47–1.92)	1.00 (0.67–1.51)	0.71 (0.47–1.07)	0.98 (0.30–3.23)
**Migration background**						
No (Ref.)	1.00	1.00	1.00	1.00	1.00	1.00
Yes	0.94 (0.70–1.26)	1.00 (0.79–1.26)	1.01 (0.71–21.43)	0.89 (0.71–1.10)	0.81 (0.65–1.00)	0.84 (0.58–1.21)
**Leadership position**						
Yes (Ref.)	1.00	1.00	1.00	1.00	1.00	1.00
No	1.21 (0.84–1.75)	1.31 (0.97–1.78)	1.80 (1.08–3.00)*	1.14 (0.87–1.51)	0.87 (0.66–1.16)	0.81 (0.47–1.42)
**School type**						
Primary school (Ref.)	1.00	1.00	1.00	1.00	1.00	1.00
Secondary general school	0.25 (0.54–1.13)	0.80 (0.36–1.77)	0.69 (0.22–2.18)	0.58 (0.27–1.26)	0.75 (0.35–1.63)	0.33 (0.76–1.44)
Intermediate school	1.21 (0.65–2.26)	0.69 (0.41–1.14)	0.58 (0.26–1.26)	0.60 (0.36–1.00)*	0.97 (0.60–1.38)	1.10 (0.47–2.80)
(vocational) grammar school	0.89 (0.51–1.57)	0.77 (0.50–1.20)	0.65 (0.36–1.25)	0.56 (0.38–0.92)*	0.90 (0.58–1.38)	0.72 (0.32–1.60)
Integrated comprehensive school	1.01 (0.54–1.90)	0.81 (0.50–1.34)	0.77 (0.36–1.61)	0.53 (0.32–0.89)*	0.85 (0.52–1.39)	0.58 (0.24–1.38)
School with several levels of qualification	1.48 (0.80–2.72)	0.56 (0.33–0.94)*	0.71 (0.33–1.51)	0.60 (0.36–0.99)*	0.83 (0.51–1.35)	0.79 (0.32–1.96)

Ref. reference group; OR, odds ratio; CI, confidence interval; *p < 0.05; **p < 0.01; NAV, navigation skills; IS, information searching; AC, adding content; ER, evaluating reliability; DR, determining relevance; PP, protecting privacy.

## 4 Discussion

To our knowledge, this is the first study examining DHL of schoolteachers. Regarding the first research question, our results show a limited DHL for 38.0% of the teachers from this study. While teachers reported fewer difficulties in DHL compared to the German general population (24.0%) difficulties are higher than those reported in a study on HL of school principals (29.0%), and in between other studies on HL of teachers (32.5–50.0%) (44, 45). At the subscale level, teachers rated the items from *operational skills* and *navigation skills* easiest, with only 0.4 to 14.3% reporting difficulties in these areas (see Table 3). This result complements previous findings using the DHLI among the general German population (58). The items rated most difficult by teachers in this study were those from the *protecting privacy* subscale, exceeding 50.0% for three of five items. This may indicate limited capabilities of the respondents, but it may also be a symptom of our modern complex media environment, and an information diversity that can be difficult to navigate (4). For example, while in a web-based study on data protection among the German population, around 64.0% of respondents felt able to maintain control over their “digital self,” equally almost 54.0% believed that they could not protect themselves from data misuse anyway if it happened (59).

Of the six binary regression models, only *navigation skills* showed significant associations with a sociodemographic factor, all other models were not significant. All had appropriate model quality as indicated by the Hosmer-Lemeshow-Test, while the level of sensitivity and specificity for three models (*navigation skills*, *information searching*, *adding content*) might indicate a limited explanatory power (see Table 1). In this regard, further individual and school-level factors and their potential association with DHL should be investigated in the future.

In our study, although not significant, male teachers displayed a higher ability in *protecting privacy*. It should be noted that in the before mentioned meta-analysis of Estrela et al. (49) the results indicated that sex does not appear to be a significant determinant of DHL. However, one reason for the sex-specific differences with *protecting privacy* in this context could also be that female teachers are more cautious about disclosing their data, while male respondents see their ability to do so as better, i.e., less problematic (60, 61). Whether this was the case in this study could not be investigated further, but in other studies there was a tendency for women to have higher concerns in relation to protecting privacy than men (62), also when using e-health services (63). Although the model was not significant, female teachers displayed a higher ability in *adding content*, which may be because women still tend to be more engaged with “health promotion, prevention and health-care measures” (4, 46) for themselves and others (38, 64). Through these interactions, they may have more reasons to judge their ability or difficulties with *adding content* related to health. Regarding the established associations with age (50), this study also found that higher age is associated with limited DHL in the dimension of *navigation skills*. Except for teachers under 40, all age groups were most likely to have limited *navigation skills*. This result may be complemented by research on general digital literacy of teachers, where a trend toward younger teachers possessing better technical skills tends to emerge (65–68). The higher competence of teachers under 40 could therefore be due to the fact that they are used to navigating on and between digital platforms and devices, as some of them could be considered digital natives.

In one of the other non-significant models, it was found that teachers who are not in a leadership role tend to be less able to write, post and share information (*adding content*) on health on digital platforms, in messengers or emails. In the context of the pandemic, school leaders had to deal more often with health-related information to manage the school during the pandemic on a day-to-day basis (45, 69). Thus, teachers with leadership roles may have had more opportunities to either train or judge their ability. Another result was that teachers from different types of schools tend to have varying degrees of difficulties regarding various aspects of DHL. This could, among other things, be associated with the different subject levels and scopes of the qualification phase of the respective school type or the respective school (media)concept, which therefore require different competence levels (e.g., digital literacy) of the teachers. Here teachers from primary schools and secondary general schools tend to report more difficulties with *evaluating reliability* than teachers from intermediate schools, (vocational) grammar schools, integrated comprehensive school, and schools with multiple courses of education. As findings in adults indicate a high proportion of limited DHL (13), an early promotion of DHL in primary schools would be important (4). Based on the Digital Competence Framework for Citizens (DigComp) of the EU the German federal states have committed themselves that beginning with the school year 2018/2019 all children enrolled in primary school (or secondary level I) will receive media competency training till the end of their school time (69); while emphasizing the necessity of promoting these abilities from the start of primary school (70). The German primary school association (Grundschulverband e.V.) pointed out that there are still open questions about how these models can be implemented in primary school (e.g., into the curricula), even if there are already ideas for implementation in the classroom (70). Thus, for primary schools the teachers might be less likely to possess sufficient ability in *evaluating reliability*, as they are not confronted with teaching this dimension in their day-to-day life as a teacher. Another finding from this study was that teachers from schools with multiple educational programs had a higher tendency for a sufficient ability in *information searching*. As most previous studies on HL differ only between primary and secondary schools, these type of school differences should be further examined. Given that individuals with different HL needs require different strategies for “engagement, education, and service delivery” (71), the question nevertheless arises as to whether it would be helpful to provide, for example, school-form-specific DHL training for teachers to best support them and their pupils with their DHL dimensions.

## 5 Strengths and limitations

Strengths of the study are the large sample, that both primary and secondary teachers are included, and that teachers were widely recruited, via online and postal invitations. One innovation that this article brings to DHL research is the expansion of the item pool of the *protecting privacy* subscale, which increased the Cronbach’s alpha from α = 0.57 to α = 0.82. This may help further research in incorporating and expanding our knowledge of teachers’ and the general populations’ ability to protect their and other’s privacy and data. The following limitations of the study should be considered when interpreting the results. When comparing the results with those of other studies, it should be borne in mind that the state of research is very limited and that the (that the few studies) few studies come from countries with different education systems. In the future, more studies are needed, especially from Europe. Second, we agree with van der Vaart and Drossaert that a web-based questionnaire has a potential bias, as it is more likely to reach teachers who use the Internet or digital devices more often (5). Furthermore, the DHLI is a self-assessment, the results could be influenced by social desirability, although there are suggestions that these effects rather take place during interviews (72). Third, a non-representative sample of teachers from Germany was used, thus, the results cannot be generalized. However, as highlighted earlier, certain similarities were found in this study, such as the percentage of women participating compared to the total number of teachers in Germany, or the 40–49 and 60+ age groups being similarly represented. Nevertheless, as there was little differentiation possible in the lower age groups, future studies should focus on recruiting younger participants, i.e., student or trainee teachers. A fourth limitation lies within the cross-sectional design, through which no statements about causality of the associations are possible. Fifth, cell frequencies in some variables turned out to be small in some cases, which is why the FET or Monte Carlo simulations were used. In future studies sufficient sample sizes should be realized in order to examine differences, e.g., with regard to the type of school. Consequently, a meaningful next step should be a representative study of German teachers to assess their DHL. Sixth, the examined predictors may not adequately explain differences, as only one binary regression model showed significance and as indicated by the sensitivity and specificity levels of *navigation skills*, *information searching* and *adding content* and the low R^2^ values of all models. Finally, besides its benefits, it has been argued that binary regression analyses can lead to loss of information and to underestimation of the extent of outcome variation between groups (73). Linear regression analyses with continuous variables could help to verify results, which, however, require continuous data.

## 6 Further research

Given our findings, a closer examination of age differences, with a particular focus on younger teachers as digital natives, may be of interest. Building on the relational nature of HL, the relevance of other individual and school-contextual factors could be examined both quantitatively and qualitatively. Examples of contextual factors and their association with a teacher’s DHL are the overall school culture, (innovation)climate, and support from school leadership, the availability and use of information and communication technologies (ICT) in the school, and the general attitude and level of integration of DHL within a school. Examples of socio-demographic factors that could be investigated are household composition, such as marital status and the number of children under care, or engagement in caregiving for others (49). It can be assumed that teachers who are in a relationship or have a care responsibility engage in surrogate health information seeking (74), its potential influence on a person’s DHL level could be further investigated. Another aspect that could be investigated are the associations between DHL and indicators of (mental) health, which have already been proven for general HL (4). If such associations are found, strong arguments could be made for the need for interventions to promote DHL, which in turn would have to be evaluated for their effectiveness on (mental) health outcomes.

The number of responses for items related to writing and sharing health-related digital messages, (subscales *adding content* and *protecting privacy*), decreased to as low as 30%. This suggests a need for further research to find out if teachers do experience the need to enhance their skills in writing, posting, and sharing health-related messages online. It could be explored whether this decrease in responses is related to the phrases used here “messages around the topic of health,” whether teachers did not find themselves in the item framing. A qualitative approach might be specifically useful in assessing these research questions.

Another question is whether the level of difficulty for *protecting privacy* would have been so high if two items in the scale had not asked about the unintentional and intentional disclosure of private data of oneself or others together. After all, it is possible that in some situations people deliberately disclose their information online without there being a direct security risk. A further need for research would therefore arise around the potential necessity to reframe items as separating the intentionally and unintentionally in items as “do you (intentionally or unintentionally) share your own private information (e.g., name or address)?” In order to contribute to HL theory building, future studies should examine the relationship between DHL and general HL to determine whether they are distinct, related, or similar concepts. Such insights may support the development of more precise concepts and models, and facilitate theory-informed, more sensitive measurement instruments.

## 6 Conclusion

Considering the findings in general, but also regarding the reported difficulties in *protecting privacy, determining reliability*, and *evaluating reliability,* there is a need for action to promote DHL among teachers. The necessity for this arises due to the stressful working conditions and their implications for the physical and mental health of teachers (4, 32–34). Moreover, the well-being of teachers correlates with improved academic performance of students (8, 36, 37), thereby influencing educational opportunities. This underscores another aspect of the importance of DHL-promotion of teachers. Furthermore, there is a need, as it can be assumed that teachers trained in general and digital HL can be multipliers for the implementation of health (literacy) promotion measures at their schools (38, 44–46). If these measures focus on general and digital HL promotion, they have the potential to empower children to take responsibility over their health (38). The promotion of DHL should be integrated into teacher training, as well as be promoted through the provision of further training courses on DHL for teachers (75). For the German context, DHL can be integrated into teacher training not as an extra, but as a topic-specific implementation of the media competency framework of the German federal states (69).

Our findings suggest that when promoting DHL in teachers some DHL dimensions might be more important and hence should be the focus of intervention efforts (*protecting privacy, evaluating reliability, determining relevance*); as this is where the greatest difficulties were reported at individual item level (see Table 3). With a few exceptions, the results suggest that all teachers can benefit equally from the promotion of DHL, as there were hardly any differences in the analyses. Nevertheless, it can be concluded that there is an increased need to promote navigation skills seen among all teachers over 40, who were more likely to have limited DHL.

In addition to the individual’s DHL, the information systems themselves should also be the subject of rethinking and change. As teachers’ DHL is related to the infrastructure that supports them in dealing with the complexity and demands of the digital space (20). Information providers should be urged to create information environments and offerings that transparently enable a high level of security in handling private data and provide reliable health-related information.

## Data availability statement

The raw data supporting the conclusions of this article will be made available by the authors, without undue reservation.

## Ethics statement

The studies involving humans were approved by Fulda University of Applied Sciences. The studies were conducted in accordance with the local legislation and institutional requirements. The participants provided their written informed consent to participate in this study.

## Author contributions

PR: Conceptualization, Data curation, Formal analysis, Methodology, Writing – original draft, Writing – review & editing. AH: Conceptualization, Data curation, Methodology, Writing – review & editing. LF: Conceptualization, Methodology, Writing – review & editing. LS: Methodology, Writing – review & editing. DR: Methodology, Writing – review & editing. OO: Conceptualization, Methodology, Supervision, Writing – review & editing. KD: Conceptualization, Funding acquisition, Methodology, Project administration, Supervision, Writing – review & editing.
